# Social science to accelerate coastal adaptation to sea-level rise

**DOI:** 10.1017/cft.2023.25

**Published:** 2023-08-29

**Authors:** Xénia Philippenko, Gonéri Le Cozannet

**Affiliations:** 1 BRGM, 45060 Orléans, France; 2 CNRS, LGP UMR 8591, Université Paris 1, Paris, France

**Keywords:** coastal adaptation, social sciences, sea-level rise

## Abstract

The latest IPCC report estimates that approximately 1 billion people will be at risk from coastal hazards in the near term due to coastal population increase, sea-level rise and other coastal changes. This will occur in a world that is changing rapidly due to climate change, ecosystem decline, human development and the projected transformations of the economy to meet the objectives of the Paris Agreement. In this context, social sciences provide a pivotal perspective to coastal adaptation, for example, while assessing barriers and opportunities across scales, from local to global. This scoping review explores how social sciences support coastal adaptation. We show that Political Sciences, Economics, Sociology and Geography are already supporting coastal adaptation. Yet, scientific fields such as legal sciences, psychology, history and archaeology as well as anthropology and ethnography are less developed in the context of coastal adaptation to sea-level rise. New research avenues could also integrate education, media and communication research and aim at truly interdisciplinary studies linking different branches of social sciences with coastal science and climate services. This effort could help moving from a coastal adaptation often focused on coastal engineering protection to a broader vision of coastal resilient development, also addressing the challenges of mitigation, sustainable development and coastal ecosystem decline.

## Graphical abstract

**Figure:**
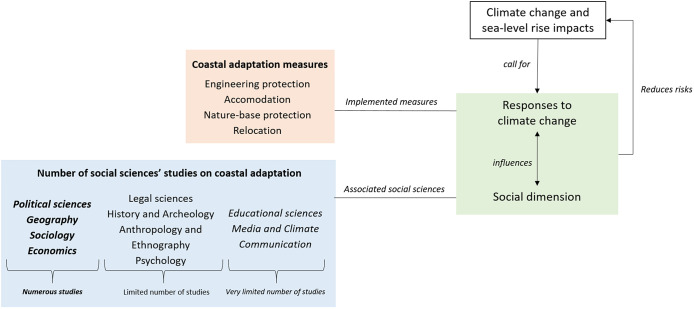

## Impact statement

Coastal adaptation is a major challenge for todays’ and future coastal communities due to ongoing and future sea-level rise. Coastal researchers and practitioners are exploring idealised coastal adaptation pathways, assuming an effective implementation of a large panel of solutions across space and time scales. Yet, empirical studies show that the implementation of adaptation is often lagging behind plans or expectations. Social sciences can help enabling coastal adaptation by providing a clearer picture of adaptation barriers and opportunities across scales, from local to global. Our scoping review shows that the contribution of social sciences applied to coastal adaptation is currently limited in scope and geographical coverage: the literature is dominated by political sciences, economics, sociology and geography. Legal sciences, psychology, history and archaeology, anthropology and ethnography, education, media and communication research can support adaptation, but they are currently providing less coastal adaptation applications. We suggest to expand the scope of social research supporting coastal adaptation, with specific attention to geographical coverage, coastal context and interdisciplinarity. Because every coastal location and community is different, no unique solution can be delivered or replicated across coastal regions in response to sea-level rise. We suggest that a global social research effort involving coastal stakeholders, exposed communities, climate services providers and scientists can support coastal adaptation efficiently. This could support communities engaging into broader social and economic transformations allowing to meet the Sustainable Development Goals in coastal areas and achieve coastal resilient development.

## Introduction

Coastal zones have changed drastically over the last centuries due to natural processes and human interventions driven by population growth, economic development and new land use practices (Brown et al., 2014). Since a few decades, climate change has increasingly becoming a major driver of change. For example, early impacts such as high-tide flooding are increasingly being reported (IPCC, 2022). Yet these events are only the onset of much larger changes along the global coastlines, as it is already well established that sea levels are committed to rise between 0.5 and 7 m, and potentially up to 15 m by 2,300 depending on future greenhouse gas emissions and the velocity of ice-sheets melting (IPCC, 2021).

**Figure 1.**
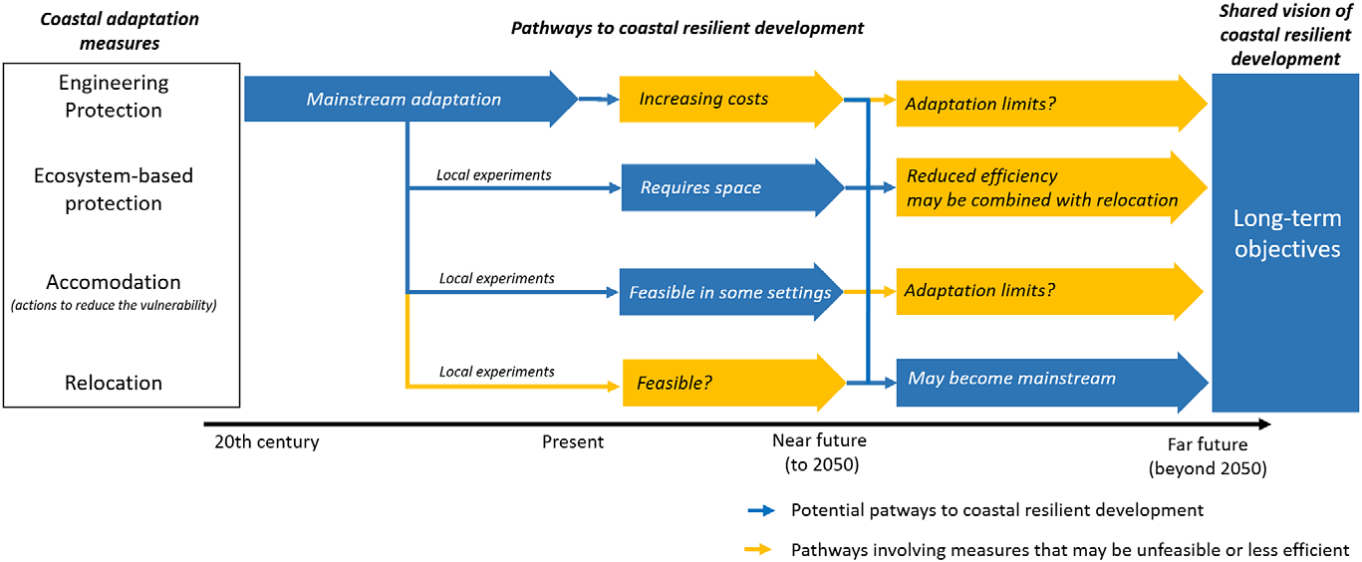
Idealised adaptation pathway leading to coastal resilient development, based on typical settings in Europe. During the 20th century, engineering protection has been the mainstream coastal risk prevention measure. This measure is becoming increasingly costly and may face adaptation limits when sea-level rise exceeds some site-specific thresholds or rates. New measures such as ecosystem-based protection or accommodation (actions to reduce vulnerability, for example, raising houses, developing early warning systems…) are being increasingly experimented locally. The feasibility of relocation is often limited today due to social and economic interest. Yet relocation may become mainstream and may be part of coastal resilient development in the future above site-specific sea levels. Figure inspired by Bednar-Friedl et al. (2022) and IPCC (2022).

Such large changes in sea levels will fundamentally change coastal socio-ecosystems worldwide. In this context, an important challenge for coastal stakeholders is to agree on long-term objectives and to identify potential pathways to achieve them. For example, coastal stakeholders may anticipate relocation on the long term, while developing some coastal protections now in order to operate some infrastructure for a few more decades (Figure 1). Such long-term objectives can contribute to climate-resilient development in coastal areas where it minimises the risks from climate change through mitigation and adaptation (Schipper et al., 2022), while also achieving sustainable development goals and reducing biodiversity losses (hereafter: coastal resilient development).

In an optimistic perspective, it can be assumed that adaptation will be implemented in an optimal way. Such optimistic postures have been studied by psychology (Lammel et al., 2013) and observed in the population (Philippenko et al., 2021). Strategies to achieve coastal resilient development can be spelt out in adaptation pathways (Haasnoot et al., 2013). From an engineering perspective, adaptation pathways have proven effective to identify when different options can be planned and implemented (Ranger et al., 2013). Yet, the fact that an adaptation pathway is efficient from an engineering point of view does not mean that it will receive public and institutional support. On the contrary, empirical evidence shows that social barriers very often explain the lack of implementation of coastal adaptation (Hinkel et al., 2018). Consequently, current adaptation is rarely transformational, that is, engaging into a pathway that may lead to coastal resilient in the long term (Figure 1; Bednar-Friedl et al., 2022). Hence, engaging into a pathway leading to coastal resilient development will require overcoming social and institutional barriers (Schipper et al., 2022).

In this context, social sciences can play a key role. Social sciences can be defined as the fields studying human societies, the way people live, the social interactions between individuals or groups and their environment, which include the study of culture, values, behaviours, policy and management, all crucial elements for adaptation to climate change (Weaver et al., 2014; Victor, 2015). They developed as an academic scientific field in the 19th century (Ross, 1993, 2003) as they emerged from older research fields, such as history, philosophy and natural history. Their development was first carried out through learned societies and some pioneer’s figures, like Alexis de Tocqueville, Alexander von Humboldt, Auguste Comte, Karl Marx and Adam Smith. Philosophy plays an important role in the process of separating and classifying different social disciplines (Moon and Blackman, 2014; Benton and Craib, 2023) – several of the pioneering figures are philosophers –, and philosophy will remain one of the ways in which the social sciences reflect on themselves over the decades. By the end of the 19th century, social sciences began to structure and distinguish themselves from one another, developing their own methods and research topics. They gather around institutions, journals such as the Annales de Géographie in France, schools of thought like the Chicago School in the United States, under the impulse of some major figures, like Emile Durkheim, Sigmund Freud, Max Weber and Franz Boaz. In the second half of the 20th century, social sciences expanded and specialised, generating multiple currents within the disciplines and giving rise to new fields of research, led by numerous researchers like Michel Foucault, Hannah Arenth, Claude Levi-Strauss, Pierre Bourdieu, Amartya Sen, Paul Lazarsfeld and many others.

Nowadays, there is no strict classification of social sciences, particularly in what distinguishes them from the human sciences (Schmaus, 2007; Guns et al., 2018; Vancauwenbergh and Poelmans, 2019; Eykens et al., 2021; Sīle et al., 2021). A broad classification could be given, considering the human sciences as centred on the individual and the social sciences as centred on social groups and societies. Here we rely on the commonly used Revised Field of Science and Technology (FOS) classification of the Organisation for Economic Co-operation and Development (OECD) to conduct studies (OECD, 2007). According to this classification, social sciences include Psychology, Economics and Business, Educational Sciences, Sociology, Law, Political Science, Geography and Media and Communication (OECD, 2007). We also add to this classification Ethnography and Anthropology, and History, which are classified by the OECD as Humanities but which we consider part of the social sciences, as they study the way human societies and groups structured themselves.

In this article, we explore how social research can enable coastal adaptation, and, more broadly, climate-resilient development in coastal zones. We carried out a scoping review, based on a search, selection and assessment of the existing literature using a keyword search on Web of Sciences (See details in Supplementary Material S1) and completing this with additional studies, for example, from the IPCC AR6 report. Because of this methodological choice and of the perimeter of a scoping review, we acknowledge that a large part of the literature in certain disciplines has been left out, including social studies published in languages other than English. Furthermore, the results depend on the specific words used in the literature and within our keyword search procedure. Despite these limitations, our work makes it possible to better characterise the type of scientific material that can be used in international reports such as the IPCC.

This review is organised as follows: Section ‘Current and potential role of social science in enabling coastal adaptation’ reviews existing studies involving social sciences in the context of coastal adaptation to sea-level rise. This section identifies branches of social sciences where there are numerous studies on coastal adaptation to sea-level rise, those that are characterised by limited number of studies, and finally those with a very limited number of studies available (Supplementary Material S2 and Tables 1–3). In section ‘Discussion’, we discuss the findings from the review and suggest new challenges. Specifically, we summarise, based on this review, important inputs from social sciences to coastal adaptation and how social science inputs to coastal adaptation can be strengthened and how a research effort could support coastal resilient development.

## Current and potential role of social science in enabling coastal adaptation

Since the first IPCC reports, social sciences have increasingly contributed to climate research (Kelly and Adger, 2000; Adger, 2003; Pelling and High, 2005; Smit and Wandel, 2006; Adger et al., 2009). Their contributions to climate science, and particularly to adaptation science, have been acknowledged by many researchers (Conrad, 2009; ISSC/UNESCO, 2013; Weaver et al., 2014; Ford et al., 2016; Holm and Winiwarter, 2017; Fouqueray and Frascaria-Lacoste, 2020). Yet, they remain insufficiently considered according to a part of the climate science community (Yearley, 2009; Weaver et al., 2014; Victor, 2015).

**Figure 2.**
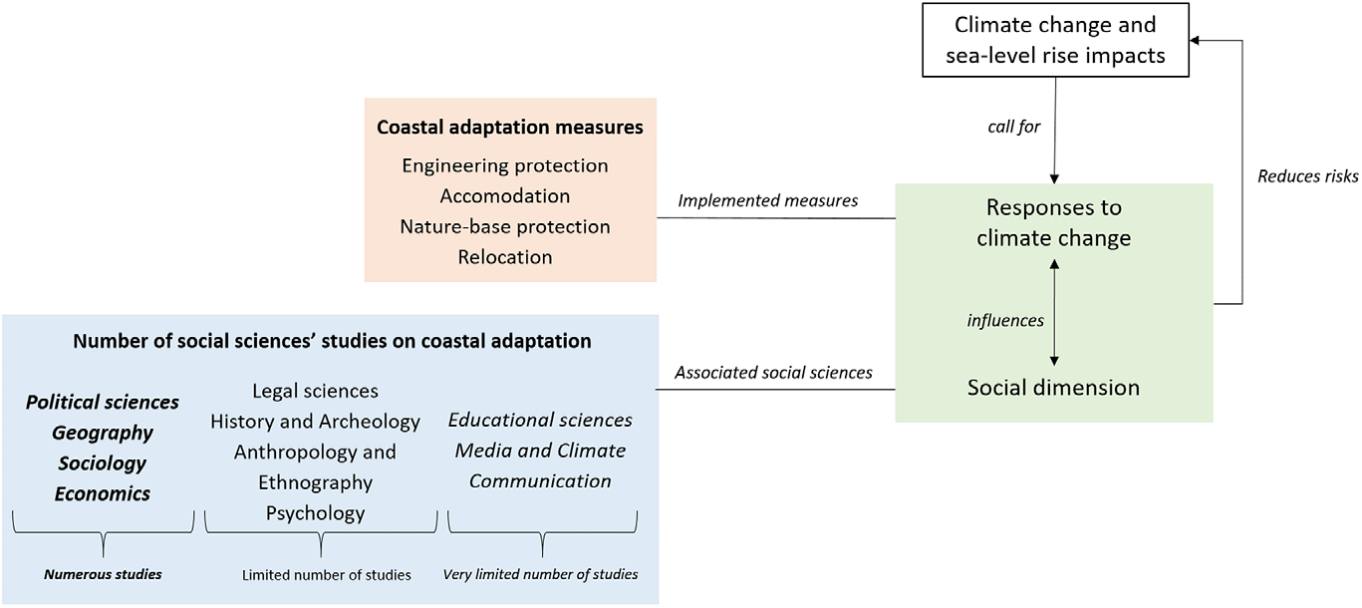
Social sciences in the context of coastal adaptation. The figure shows how social sciences are positioned in the context of coastal adaptation implementation. It highlights the unequal number of studies available in the peer-reviewed literature depending on each branch of social sciences.

The IPCC AR6 partly responds to this criticism by developing narratives that consider socio-economic challenges: it considers the social dimension of risk, indigenous knowledge, climate justice and equity, points out the importance of socio-economic and behavioural conditions to enable adaptation and gives an increasing space to governance issues and challenges (IPCC, 2022). Nevertheless, economics and governance remain a prominent entry point for social sciences in IPCC reports. This suggests that social sciences could make a greater contribution to climate change adaptation research and operations (Figure 2).

Based on the classification presented above, we explore how the 10 social science fields identified in section ‘Introduction’ support coastal adaptation: Tables 1–3 summarise for each of these 10 disciplines the type of studies and analysis they can provide and present their contributions to enable coastal adaptation to sea-level rise. When articles provide information on their theoretical foundations, Tables 1–3 also distinguish between applied and theory-driven studies, noting that this information is not always provided, as reported by Kuhlicke et al. (2023).

Tables 1–3 show that the 10 social science disciplinary fields identified in section ‘Introduction’ based on OECD (2007) are not equally contributing to coastal adaptation, as shown in Figure 2 and Supplementary Material S2 as well. Some disciplines or some currents within these disciplines remain under-represented, such as Psychology or History or Communication and Educational Sciences, while others are the subject of a more extensive literature, such as Political Sciences or Economic analysis (Supplementary Material S2). In the remainder of this section, we examine successively social sciences for which numerous studies on coastal adaptation to sea-level rise are available, that is, more than 50 to several hundred studies depending on the discipline (section ‘Social sciences delivering numerous studies on coastal adaptation to sea-level rise’), those that are characterised by a more limited number of studies, that is, around 20 studies per discipline (section ‘Social sciences delivering a more limited number of studies on coastal adaptation to sea-level rise’) and finally those for which only a very limited number of studies can be identified, that is, less than 20 studies available per discipline (section ‘Social sciences delivering a very limited number of studies on coastal adaptation to sea-level rise’).

### Social sciences delivering numerous studies on coastal adaptation to sea-level rise

Local or global conditions enabling coastal adaptation are explored by a significant and growing number of studies, notably in *Political Sciences* (Table 1). This literature has a particular focus on decision-making and assesses local, national and sub-national coastal adaptation policies (Aguiar et al., 2018; Reckien et al., 2018; Olazabal et al., 2019; Gussmann and Hinkel, 2021), drivers of adaptation (Simonet and Leseur, 2019; Gussmann and Hinkel, 2020; Landauer et al., 2021), needs, priorities and perspectives of stakeholders (Frazier et al., 2010; Hinkel et al., 2019; Terorotua et al., 2020; van Ginkel et al., 2020), the question of citizen engagement (Jarvis et al., 2015; Wamsler et al., 2020; Areia et al., 2022), the articulation of scales of governance across local, national or sub-national institutions (Agrawal, 2008; Petzold and Magnan, 2019; Petzold and Ratter, 2019; Therville et al., 2019; Rocle et al., 2021), global or local barriers to adaptation (Eisenack et al., 2014; Waters et al., 2014; Simonet and Leseur, 2019; Whitney and Ban, 2019; Lee et al., 2022), as well as decision-making under uncertainty (Walker et al., 2013; Kwakkel et al., 2016; Lawrence et al., 2019; Lawrence et al., 2020). The latter is a particularly important issue in coastal areas, where stakeholders have to deal with a very wide range of sea-level rise projections (Fox-Kemper et al., 2021). New themes are developing in the field of Political Sciences, such as the study of the science/society interface, in particular the study of the contribution of climate services for a better decision-making (Hewitt et al., 2017; Kopp et al., 2019; Bisaro et al., 2021; Lawrence et al., 2021; Simm et al., 2021), and other themes are still emerging, such as the question gender equity (Vasseur et al., 2015; McLeod et al., 2018; Malik et al., 2021; Prakash et al., 2022), or the inclusion of disabled people (Barua and Molla, 2019; Molla et al., 2019) or religious and ethnic minorities (Ahmed and Ampadu, 2019; Sen et al., 2020) within public adaptation policies.

**Table 1.** Fields of social sciences for which numerous studies on coastal adaptation to sea-level rise can be identified (more than 70 studies)

		References (not exhaustive)				
Field of study	Type of analysis	Mainly theory-driven studies	Mainly applied studies (based on case studies)	Both applied and theory-driven studies	Reviews	Potential for enabling adaptation
Political sciences	Adaptation policies		Aguiar et al., 2018; Reckien et al., 2018; Olazabal et al., 2019; Gussmann and Hinkel, 2020			Build governance capacities to tackle complex problems; Design and facilitate tailor-made participation processes, involving stakeholders early and consistently from negotiating responses to implementation; Develop networks and linkages within and between governance scales and levels and across policy domains and sectors, to improve coordination, build trust and legitimise decisions; Keep options open to adjust responses as climate risk escalates and the contexts evolves
	Stakeholders’ needs and priorities (including for climate services)	Hinkel et al., 2019	Frazier et al., 2010; Terorotua et al., 2020	van Ginkel et al., 2020		
	Governance and institutional barriers and drivers	Eisenack et al., 2014	Waters et al., 2014; Simonet and Leseur, 2019; Whitney and Ban, 2019; Gussmann and Hinkel, 2020	Landauer et al., 2021	Lee et al., 2022	
	Adaptation decision-making, including under uncertainty	Walker et al., 2013; Kwakkel et al., 2016	Lawrence et al., 2019			
	Science/society interface (including for climate services)	Hewitt et al., 2017; Kopp et al., 2019; Bisaro et al., 2021; Simm et al., 2021		Lawrence et al., 2021		
	Participatory democracy and citizen’s engagement		Jarvis et al., 2015; Wamsler et al., 2020; Areia et al., 2022		Hügel and Davies, 2020	
	Inclusive democracy within adaptation: gender, ethnic and religious minorities, disabled people		Vasseur et al., 2015; McLeod et al., 2018; Ahmed and Ampadu, 2019; Barua and Molla, 2019; Molla et al., 2019; Sen et al., 2020; Malik et al., 2021		Prakash et al., 2022	
Geography and sociology	Consideration of equity and justice aspects in adaptation process, including gender and ethnicity’s concern		Islam, 2010; Chakraborty et al., 2014; Smith and Rhiney, 2016		Bunce and Ford, 2015; Pearse, 2017	Recognise socio-economic and political realities and prioritise vulnerability, justice and equity concerns to enable just, impactful and enduring outcomes; Facilitate the design of adaptation policies adapted to the needs and behaviours of populations; Avoid new development commitments in exposed locations; Enable managed retreat in most at-risk locations by anticipatory actions; Facilitate the design of adaptation policies adapted to the needs and behaviours of populations
	Perceptions of climate change and adaptation		O’Neill et al., 2015; Brennan et al., 2016; Coquet et al., 2018; Goeldner-Gianella et al., 2019; Lemée et al., 2020; Ruz et al., 2020		Becerra et al., 2020	
	Risk and vulnerability assessments		Boateng, 2012; Duvat et al., 2021	Balica et al., 2012; Duvat et al., 2017; Kelly and Adger, 2000	Thomas et al., 2019	
	Land-use planning for adaptation	Wedin and Wikman–Svahn, 2021	Frazier et al., 2010; Hurlimann et al., 2014; Robert and Schleyer-Lindenmann, 2021			
	Contemporary climate-driven migrations	Horton et al., 2021	King et al., 2014; Bronen, 2015; Hino et al., 2017; Duvat et al., 2022	Alexander et al., 2012; Luetz and Merson, 2020	O’Donnell, 2022	
	Acceptability of adaptation solutions and processes		Goeldner-Gianella et al., 2015; Dachary-Bernard et al., 2019; Anderson et al., 2021; Philippenko et al., 2021		Anderson and Renaud, 2021; Mallette et al., 2021;	
Economics and business	Economic analysis and comparison of adaptation solutions (including Cost-Benefits Analysis)		Basco, 2015; Creach et al., 2020	André et al., 2016; Vousdoukas et al., 2020; Bachner et al., 2022		Recognise socio-economic and political realities and prioritise vulnerability, justice and equity concerns to enable just, impactful and enduring outcomes; Enable managed retreat in most at-risk locations by anticipatory actions
	Economic contribution of actors of adaptation (companies, public authorities, citizens, NGO, etc.)	Herweijer et al., 2009; Buso and Stenger, 2018; Costa et al., 2020	Islam and Walkerden, 2015		Bisaro and Hinkel, 2018	
	Consideration of economic equity and justice aspects in adaptation process		Siders, 2019; Long et al., 2022	Adelman, 2016		
	Funding conditions and framework for adaptation	Freeman, 2004; Henderson, 2018; Woodruff et al., 2020	Merrill et al., 2018; Rey-Valette et al., 2018; Moser et al., 2019; Paleari, 2019; Barraqué and Moatty, 2020; Bisaro et al., 2020	Boston et al., 2021		

These themes are also addressed by studies falling within the fields of *Geography* (Chakraborty et al., 2014; Bunce and Ford, 2015; Smith and Rhiney, 2016) and *Sociology* (Islam, 2010; Pearse, 2017). These disciplines also study representations of the climate change and of adaptation; more specifically, Geography seeks to spatialize these representations, distinguishes the differences in representations between populations and places, and sheds lights on the factors shaping these perceptions (O’Neill et al., 2015; Brennan et al., 2016; Coquet et al., 2018; Goeldner-Gianella et al., 2019; Becerra et al., 2020; Lemée et al., 2020; Ruz et al., 2020). This can facilitate the implementation of adaptation plans by considering the different needs and representations of local populations. Risk and vulnerability assessment, which has been addressed by geography for several decades, has also become central to the study of coastal adaptation (Kelly and Adger, 2000; Balica et al., 2012; Boateng, 2012; Duvat et al., 2017; Thomas et al., 2019; Duvat et al., 2021), and has become increasingly important in the IPCC reports: inequalities in vulnerability may indeed hinder adaptation. Geographers are also addressing other issues, such as spatial planning for adaptation (Frazier et al., 2010; Hurlimann et al., 2014; Robert and Schleyer-Lindenmann, 2021; Wedin and Wikman–Svahn, 2021) or the question of climate-driven migrations. The literature distinguishes internal relocation within the same space, and international migration, which pushes populations to leave their island or their coastal areas to move elsewhere (Alexander et al., 2012; King et al., 2014; Bronen, 2015; Hino et al., 2017; Luetz and Merson, 2020; Horton et al., 2021; Duvat et al., 2022; O’Donnell, 2022), creating the notion of ‘climate refugees’. In both cases, these climate-driven migrations are rarely chosen and not well accepted. Recently, studies have assessed the acceptability of these policies, as well as adaptation policies in general. Such studies can help to better understand the factors and profiles of the populations, to better implement adaptation policies (Goeldner-Gianella et al., 2015; Dachary-Bernard et al., 2019; Anderson et al., 2021; Anderson and Renaud, 2021; Mallette et al., 2021; Philippenko et al., 2021).

*Economic* analyses of adaptation solutions, in particular cost–benefit analyses, are numerous (Basco, 2015; André et al., 2016; Creach et al., 2020; Vousdoukas et al., 2020; Bachner et al., 2022). Studies on adaptation finance are developing and would benefit from further research, including a detailed assessment of the current and potential role of actors, such as businesses, insurances, Non-Governmental Organisation (NGO) and citizens (Herweijer et al., 2009; Islam and Walkerden, 2015; Bisaro and Hinkel, 2018; Buso and Stenger, 2018; Costa et al., 2020). Studies on adaptation finance can also help establishing a clearer vision of the economic opportunities and constraints in different coastal settings. For example, they make it possible to develop coastal adaptation pathways that consider equity and justice from an economic perspective (Adelman, 2016; Siders, 2019; Long et al., 2022). From this perspective, studies on the conditions for financing adaptation, on the financial framework and the more detailed financing mechanisms are essential. The number of studies relevant to these issues is growing (Merrill et al., 2018; Moser et al., 2019; Bisaro et al., 2020; Woodruff et al., 2020). For example existing mechanisms for financing relocation (Henderson, 2018; Rey-Valette et al., 2018; Boston et al., 2021; Keeler et al., 2022) or compensation following a natural disaster (Freeman, 2004; Paleari, 2019; Barraqué and Moatty, 2020) are receiving increased attention. Importantly, assessing the various aspects of the economic dimension of adaptation is increasingly recognised as essential, as it allows expensive adaptation actions such as relocation to be better anticipated (Turner et al., 2007; André et al., 2016).

### Social sciences delivering a more limited number of studies on coastal adaptation to sea-level rise

We explore in this section social sciences that are characterised by a more limited number of studies on coastal adaptation to sea-level rise. These disciplines exist since a long time, and may have contributed to research on coastal societies. However, their engagement into coastal adaptation research remains limited, based on our statistical analysis (Supplementary Material S2).

*Legal Sciences* can address numerous problems relevant to coastal adaptation, but the number of studies remains limited (Table 2). Areas of interest include the legal aspects of infrastructures relocation (Fowler et al., 2022), spatial planning and management in coastal areas (Thom, 2004; Patlis, 2005) or compensation of populations facing natural disasters (Farber, 2013). Legal studies can assess the legal provisions that exist in each country, or area of legislation to regulate the adaptation of coastal zones (Verschuuren and McDonald, 2012; O’Donnell, 2019; Oral, 2019; Schumacher, 2020). They can also explore the legal gaps that still exist in existing regulations related to coastal adaptation (Reiblich et al., 2017, 2019). The question of the interweaving of the different legislative scales – local, national and sub-national – is also a relevant research topic, as it sometimes leads to conflicts due to a misunderstanding of the different levels of legislation or to contradictions between them. One typical example is the misalignment between the local law of indigenous communities and that of the state in which these community live (Williams and Hardison, 2014; Davies, 2015). This raises the question of equity and climate justice, through the prism of legal mechanisms (Adelman, 2016; Lambert et al., 2019; Nurhidayah and McIlgorm, 2019).

**Table 2.** Fields of social sciences for which a more limited number of studies on coastal adaptation to sea-level rise can be identified (around 20 studies)

		References (not exhaustive)				
Field of study	Type of analysis	Mainly theory-driven studies	Mainly applied studies (based on case studies)	Both applied and theory-driven studies	Reviews	Potential for enabling adaptation
Legal studies	Existing legal framework relevant to coastal adaptation	Farber, 2013	Thom, 2004; Patlis, 2005; Verschuuren and McDonald, 2012; O’Donnell, 2019; Schumacher, 2020; Fowler et al., 2022	Oral, 2019		Anticipating and enabling managed retreat in some locations that are most at risks; Avoid new developments in exposed locations
	Legal gaps into adaptation implementation	Reiblich et al., 2017	Reiblich et al., 2019			
	Interweaving of different legal levels and structures, in particular between indigenous people and state legislation		Davies, 2015	Williams and Hardison, 2014		
	Consideration of equity and climate justice		Baptiste and Devonish, 2019; Lambert et al., 2019; Nurhidayah and McIlgorm, 2019	Adelman, 2016		
Ethnography and anthropology	Consideration and understanding of Indigenous Knowledge into adaptation strategies		Hiwasaki et al., 2014; Granderson, 2017; Romero Manrique et al., 2018			Strengthen community capabilities to respond to coastal hazard risk; Build shared understanding and enable locally appropriate responses through experimentation, innovation and social learning; Facilitate the design of adaptation policies adapted to the needs and behaviours of populations
	Values, faith and relationship to environment of coastal communities		Nunn et al., 2017; Bertana, 2020			
History and archaeology	Long-term analysis of human relationship and adaptation to environment	Pfister, 2010; Guedes et al., 2016		Rockman and Hritz, 2020		Adopt a long-term view but take action now; Avoid maladaptation by considering the historical, socio-economic and political context
	Understanding of historical barriers	Adamson et al., 2018	Ferdinand, 2018; Moulton and Machado, 2019; Bordner et al., 2020			
	Identification of past practices and trajectories of vulnerability in coastal areas		Orlove, 2005; Arenstam Gibbons and Nicholls, 2006; Janif et al., 2016; Athimon and Maanan, 2018; Soens, 2018; Duvat et al., 2021	Duvat et al., 2017		
Psychology	Understanding cognitive barriers and enablers of adaptation	Grothmann and Patt, 2005; Clayton et al., 2015	Bruno et al., 2021	Lammel et al., 2013		Facilitate the design of adaptation policies adapted to the needs and behaviours of populations May strengthen cognitive capabilities that facilitate social adaptation
	Understanding values as barriers and enablers of adaptation	Roser-Renouf et al., 2014				
	Understanding adaptation behaviour	Brügger et al., 2021	Bradley et al., 2020			

Research on current practices and relationships to the environment is often the purpose of *Anthropology* and *Ethnography.* The importance of these disciplines is increasingly being recognised in the literature on coastal adaptation to climate change. The latest IPCC report addresses it through the lens of indigenous communities (IPCC, 2022). Specifically, this report emphasises the importance of considering indigenous knowledge, both in the assessment of climate change and in the implementation of adaptation measures (Hiwasaki et al., 2014; Granderson, 2017; Romero Manrique et al., 2018; Mycoo et al., 2022). However, there is currently a gap of knowledge in the IPCC reports and scientific literature on coastal adaptation: for example, in the chapter on cities and settlements by the sea (Glavovic et al., 2022), only 11 occurrences of the term ‘indigeneous’ and 5 references containing this term were found. Although a various literature exists in Anthropology on climate adaptation, the number of studies applied to coastal adaptation is scarcer (Supplementary Material S2). The role of values, faith and relationship to the environment of local communities is however becoming increasingly recognised and studied for successful adaptation (Nunn et al., 2017; Bertana, 2020). Apart from the Indigenous Societies, the practices and the relationship to the environment of coastal societies, remain little studied, even though they could also bring some elements of understanding (Mazé et al., 2017).

There are still relatively few studies on coastal adaptation in *History* and *Archaeology.* Yet these two branches of Social Sciences could help better understanding the evolving relationship between societies and their coastal environment over time, the changes in this relationship and the factors behind these changes (Pfister, 2010; Guedes et al., 2016; Rockman and Hritz, 2020). This can benefit adaptation because current barriers may be historically rooted in a particular period such as colonialism (Ferdinand, 2018; Moulton and Machado, 2019; Bordner et al., 2020), in a specific relationship to the environment, or in social, economic or political lock-ins that have been established and maintained over time (Adamson et al., 2018). Understanding vulnerability trajectories over time (Orlove, 2005; Arenstam Gibbons and Nicholls, 2006; Duvat et al., 2017, 2021) and the positive and negative legacy of prevalent practices inherited from the past (Janif et al., 2016; Athimon and Maanan, 2018; Soens, 2018) makes it possible to shed light on the links between past and present situations. Strengthening the contribution of these branches of social sciences to coastal adaptation could avoid overlooking barriers and opportunities that are crucial for local coastal communities.

Part of people’s relationship to the environment is also linked to the cognitive abilities of individuals. This aspect is studied by *Psychology.* Psychology makes it possible to uncover cognitive or psychological barriers to climate change and adaptation (Grothmann and Patt, 2005; Lammel et al., 2013; Clayton et al., 2015), to better understand the role of individual and collective values on cognitive processes leading to action (Roser-Renouf et al., 2014), and to study how behaviours evolve as climate is changing and adaptation is being implemented (Bradley et al., 2020; Brügger et al., 2021). In coastal areas, psychological factors are especially relevant, for example, because the sea is often perceived by individuals as a source of positive amenities, which can create a cognitive barrier by reducing awareness of current and future coastal risks (Bruno et al., 2021). A better understanding of these elements is therefore essential for the success of adaptation plans.

As a summary, there is empirical evidence that Legal sciences, Anthropology, Ethnography, History, Archaeology and Psychology can help understanding coastal adaptation and ultimately support it. Yet, the number of studies relevant to these areas is more limited than for the branches of social sciences reviewed in section ‘Social sciences delivering numerous studies on coastal adaptation to sea-level rise’. Nevertheless, the papers reviewed in this section suggest that further developing the emerging research reviewed in section ‘Social sciences delivering a more limited number of studies on coastal adaptation to sea-level rise’ can ultimately support coastal adaptation.

### Social sciences delivering a very limited number of studies on coastal adaptation to sea-level rise

While scientific research on *Media and climate Communication* has existed for decades, it is developing and gaining in importance in recent years with the recognition that Medias contribute to shaping perceptions on climate change impacts, adaptation and mitigation (Table 3; Chen et al., 2021). This creates new research areas such as the use of social media and networks in relation with risks and disasters (Takahashi et al., 2015; Kryvasheyeu et al., 2016; Niles et al., 2019). Some studies focus on media coverage of climate change in coastal areas and adaptation solutions (Smith and Joffe, 2009; Rick et al., 2011; Schmidt et al., 2013; Jaspal and Nerlich, 2014; Painter, 2015; Ford and King, 2015; Akerlof et al., 2017), on the role that media play on people’s representations (Joffe and Orfali, 2005; Olausson, 2011; Goeldner-Gianella et al., 2019) and their influence on adaptation behaviour (Bowden et al., 2021). For example, it is now well established that Medias can help understanding climate change through narratives explaining the links between climate change and extreme phenomena, many of which affect coastal areas, such as storms, cyclones or chronic flooding at high tide.

**Table 3.** Fields of social sciences are characterised by a very limited number of studies on coastal adaptation to sea-level rise (less than 20 studies)

		References (not exhaustive)				Potential for enabling adaptation
Field of study	Type of analysis	Mainly theory-driven studies	Mainly applied studies (based on case studies)	Both applied and theory-driven studies	Reviews	
Media and communication studies	People’s use of media during risks, disasters and the adaptation process		Takahashi et al., 2015; Kryvasheyeu et al., 2016; Niles et al., 2019			Build shared understanding and enable locally appropriate responses through experimentation, innovation and social learning
	Media coverage of climate change in coastal areas and adaptation solutions		Smith and Joffe, 2009; Rick et al., 2011; Schmidt et al., 2013; Jaspal and Nerlich, 2014; Ford and King, 2015; Painter, 2015; Akerlof et al., 2017			
	Role of media on climate change representations	Joffe and Orfali, 2005	Olausson, 2011; Goeldner-Gianella et al., 2019			
	Influence of media on adaptation action		Bowden et al., 2021			
Educational studies	Role of education in understanding climate change and enabling adaptation	Cooper, 2011	Damerell et al., 2013; Muttarak and Lutz, 2014; Demant-Poort and Berger, 2021; Kolenatý et al., 2022	Borde et al., 2020		Strengthen community capabilities to respond to coastal hazard risk; Build shared understanding and enable locally appropriate responses through experimentation, innovation and social learning
	Evaluation and integrating social sciences in climate literacy	Shwom et al., 2017				
	Studies on the science/society interface (climate services, educational resources, serious game…)		Mouaheb et al., 2012; Rumore et al., 2016; Fleming et al., 2020; Neset et al., 2020		Flood et al., 2018	

*Educational Studies* are complementary to media and communication sciences. Like media and communication, education plays a major role in understanding climate change and enabling adaptation (Cooper, 2011; Damerell et al., 2013; Muttarak and Lutz, 2014; Borde et al., 2020; Demant-Poort and Berger, 2021; Kolenatý et al., 2022). Importantly, a stronger integration of social sciences in environmental science school and academic curricula could be beneficial and avoid that solutions mostly focused solely on engineering and physical sciences (Shwom et al., 2017). A new field of research is also developing in this discipline is the study of the science-society interface and the resources that are developed in this framework, such as climate services, educational resources or serious games (Mouaheb et al., 2012; Rumore et al., 2016; Flood et al., 2018; Fleming et al., 2020; Neset et al., 2020).

To summarise, research on Media, Climate Communication and Education is a new research avenue that is developing as concerns are growing regarding climate change and its coastal impacts, and as Medias, teachers, science educators and academics are becoming increasingly aware of their role in shaping representations and providing knowledge on climate change. Importantly, this research supports the uptake of climate literacy in the wider public, which can improve the responses, as reminded in the latest IPCC reports (IPCC, 2021, 2022). More interdisciplinary research assessing the interactions between Media, Climate Communication and Education and adaptation to climate change could be beneficial to accelerate adaptation, including in coastal areas.

Finally, we point out that each disciplinary field within social sciences can explore different dimensions of coastal adaptation in a complementary way. As seen throughout this study, some dimensions of coastal adaptation research have been even addressed by several disciplines. One example is the topic of relocation in coastal areas, which has been addressed by studies falling within areas such as by Economics (André et al., 2016; Dachary-Bernard et al., 2019; Creach et al., 2020; Keeler et al., 2022), Political Sciences (Gussmann and Hinkel, 2020), Legal Science (Fowler et al., 2022), Anthropology (Janif et al., 2016; Bertana, 2020) or Geography (Alexander et al., 2012; Duvat et al., 2022).

## Discussion

### Key inputs from social sciences to coastal adaptation

Not surprisingly, section ‘Current and potential role of social science in enabling coastal adaptation’ and Tables 1–3 confirm that social sciences can cover a very high number of topics relevant to coastal adaptation research. It also reminds that social research not only improves our understanding of behaviours, prevalent practices, social norms and representations of individuals and social groups, but can also support coastal adaptation in practice (see fourth column in Tables 1–3). For example, a social science project on coastal adaptation can support practitioners responsible for defining or implementing coastal adaptation policies that consider the needs and priorities of coastal communities, and not only technical or economic criteria. The potential benefits for coastal adaptation practitioners are to move towards greater efficiency and to promote stable and long-term adaptation policies that are just and impactful. Social sciences can also promote the engagement of citizens in participatory democracy, which improves the trust of individuals in the adaptation process and facilitates the implementation of adaptation policies (Table 1; Hügel and Davies, 2020). Social sciences also approach adaptation across the world, and therefore underline the diversity of socio-economic, ecological and political-institutional contexts, recalling that no unique coastal adaptation solution or pathway could be replicated or adapted across coastal regions and localities. Hence, our review confirms that social science on coastal adaptation can bring important benefits on the ground.

### Strengthening social science inputs to coastal adaptation

Yet, some disciplines within social sciences remain insufficiently considered in coastal adaptation despite clear evidence that they can bring social benefits. For example, Anthropology and Psychology could be much more developed and integrated into existing research programmes (section ‘Social sciences delivering a more limited number of studies on coastal adaptation to sea-level rise’), including in new programmes focusing mainly on physical sciences and service development. Interdisciplinary research has been conducted, for example, on coastal adaptation geography and economy (Creach et al., 2020; Long et al., 2022) or political science and economy (van Ginkel et al., 2020; Woodruff et al., 2020), yet the majority of studies involve only one discipline, or two at best (Supplementary Material S3). Even more, interdisciplinarity could be promoted and ultimately benefit the practice of coastal adaptation. Finally, it is not clear to what extent the literature on social sciences applied to coastal adaptation is considered by public policies and decision-makers, beyond those already engaged with social scientists. On the contrary, the IPCC report suggests that top-down engineering solutions still dominate, at least in regions such as Europe, despite their potential to damage coastal ecosystems, create lock-ins or perpetuate a commitment to maintain coastal defences on the long term (IPCC, 2022).

This limited consideration of social sciences in the practice of coastal adaptation might be due to the prevalent work practices of many coastal adaptation decision-makers, which often have a coastal engineering background and do not necessarily all know the benefit of a social approach. The strong focus of large research programmes on climate services development, at least in Europe, is efficient to inform coastal adaptation decisions quickly and efficiently (Le Cozannet et al., 2017), but this entry point does not ease the integration of social sciences beyond those directly concerned with existing risk management decisions. Furthermore, existing management decisions are most often incremental, while IPCC reports raise the need for transformational adaptation to respond to climate change (Bednar-Friedl et al., 2022). Hence, there is a risk to miss the transformative dimension of adaptation. One potential interdisciplinary research avenue to resolve this issue could be to develop decision-making tools and methods that integrate socio-economic transformations in a climate service informing transparently about the social feasibility and co-benefits of each coastal adaptation solution.

The development of social science applied to coastal adaptation should consider the spatial imbalance that currently exists in the scientific literature. Many studies are focused on Europe, North America, Southeast Asia or Small Islands as a specific geographic entity facing its own challenges due to climate change, although studies have expanded to other geographical areas in recent years (Anderson and Renaud, 2021; Mallette et al., 2021; Cabana et al., 2023). Social research applied to coastal adaptation outside the Western world is still underdeveloped despite their highly diversified history, cultures and values. Developing social research in different coastal environments and in different types of coastal management units (sandy, muddy, cliffed or artificial coasts and estuaries) can help reveal contrasted coastal adaptation and development opportunities. For example, sea-level rise questions about the current land use and can ultimately lead to territorial reconfigurations in coastal areas that are already developed. Yet in developing coasts and countries such as in Africa and Asia, there is the opportunity to consider sea level rise in the design of cities and in land-use planning now in order to avoid lock-ins in the future. Indeed, the IPPC Sixth Synthesis Report (Lee et al., 2022) shows that until 2040, exposure to coastal flooding will not only increase due to sea-level rise, but also due to development in coastal areas. For example, they estimate that an additional 1.25 million people in Africa and 7 million in Asia will be exposed to coastal flooding due to sea-level rise only. If we add the additional population caused by population growth and coastal development, the number of additional people exposed to flooding increases to 2.29 million in Africa and 16.39 million in Asia. This assumes a ‘middle of the road’ Socio-Economic Pathway (SSP2). Yet, population growth in the coming two decades are largely locked in already and display limited differences depending on Socio-Economic Pathways at these broad scales (Merkens et al., 2016). This raises serious concerns regarding projected trends in coastal land use in the context of ongoing sea-level rise, at least in Africa and Asia. This illustrates that coastal adaptation planning can benefit from taking into account socio-economic projections such as those derived from the Socio-Economic Pathways.

### Enabling coastal resilient development

**Figure 3.**
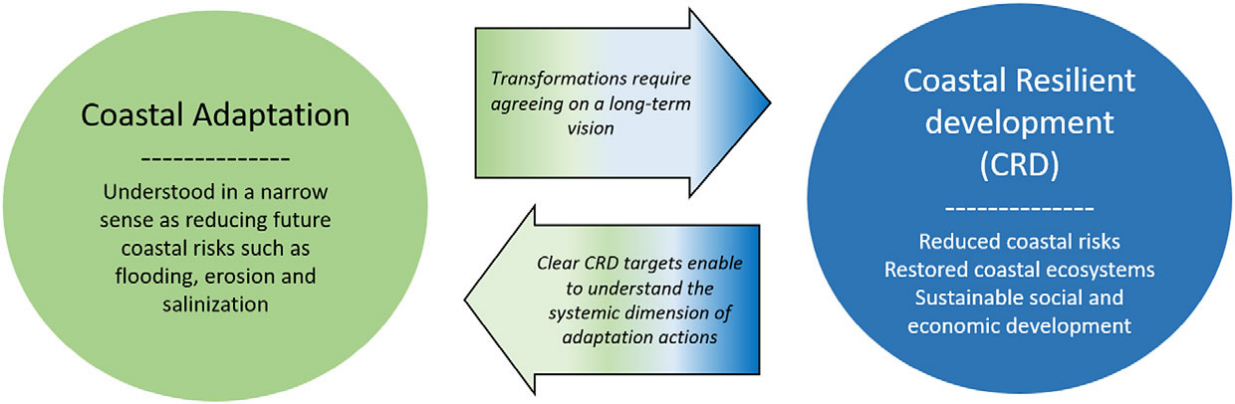
Illustrative transition from a coastal adaptation focused mainly on limiting future coastal risks to a coastal resilient development considering other economic, social and ecosystemic transformations in coastal areas. Social sciences can help in expanding the scope of coastal transformations considered at different decision levels.

Climate change and sea-level rise projections clearly show that current coastal development needs transformations to limit future coastal risks, restore coastal ecosystems, stimulate economic activities and ensure wellbeing (IPCC, 2022). These transformations go far beyond the sole topic of coastal adaptation and can be referred to as coastal resilient development (Figure 3). In the IPCC report, it corresponds to a situation where sustainable development goals have been achieved, including mitigation of and adaptation to climate change (IPCC, 2022). Enabling such a resilient coastal development implies agreeing on a long-term vision that considers the decades and centuries of sea-level rise to come, for example when designing new coastal infrastructures such as coastal nuclear plants or estuarine barriers. It also requires agreeing on what it means to achieve sustainable development goals in each coastal zones in terms of economic activities, land use and coastal protection. Defining and agreeing on such a long-term vision is essential: coasts will be facing systemic transformation, including coastal ecosystems changes, energy transition implications, transformations in sectors such as fisheries, urban planning, port and coastal industries, and so forth.

Such long-term visions are rarely explicitly stated. In many places, there is a high social demand for protection, so that the default approach often consists in raising coastal protection (Mallette et al., 2021). This type of approach comes along with trade-offs, for example in terms of costs, biodiversity conservation and tourism amenities. Softer coastal management approaches that leaves more space to coastal ecosystems are slowly emerging in Europe though their benefits are known since decades (e.g., www.eurosion.org). For example in France, the Conservation Agency is experimenting the renaturation of coastal sites and a retreat of coastal defences inland in close collaboration with other coastal communities (Bazin and Olivry, 2017). Locally, a long-term vision may include a long-term commitment to coastal protection in some places and a progressive relocation and renaturation project in other areas. Whatever the objectives, we argue that a clear and transparent process for defining long-term objectives is essential to create trust in the process of coastal adaptation and to enable coastal resilient development. Once the long-term vision is defined, pathways to achieve them can be debated (Haasnoot et al., 2013).

Social sciences will not deliver this vision, but they can help coastal stakeholders to formalise them, propose opportunities and potential ways forwards revealed by various disciplines of social sciences. For territories that are considering alternatives to engineering protection, social sciences can be crucial: they can help raise awareness, they can study and develop the acceptability of adaptation alternatives, they can facilitate dialogue between stakeholders or propose a historical perspective to the occupation and evolution of the territory. In summary, we argue that social sciences are an essential part of science to enable coastal adaptation and resilient coastal development. The local benefits of this research are obvious for coastal communities as it can help shaping and fostering climate-resilient development locally.

## Conclusion and perspectives

This scoping review highlights how social sciences could help enabling coastal adaptation by providing a clearer picture of adaptation barriers and opportunities across scales, from local to global. This includes analyses of populations and stakeholders priorities, needs, perceptions and adaptation capacity, considering also ethical aspects such as equity and inclusiveness. We identify branches of social sciences already contributing significantly to coastal adaptation, such as Political Sciences, Economics, Sociology and Geography, and other that are developing but still lagging behind such as Legal Sciences, Psychology, History and Archaeology as well as Anthropology and Ethnography. New research could better integrate Educational, Media and Communication Sciences, and aim at better integrating the various branches of social sciences with coastal engineering, geomorphology, environmental and ecological sciences. We show that this research can support adaptation (Tables 1–3) and may help moving away from a practice of coastal adaptation often focused on engineering protection.

Due to the wide variety of biophysical and social contexts, there is no unique coastal adaptation solution or pathway that could be replicated or adapted across coastal regions and localities. Hence, we argue that a global social research effort, well connected to coastal stakeholders, exposed populations, climate services providers, and other relevant scientific areas could support coastal adaptation efficiently.

Our scoping review is a first effort to explore how different fields of social sciences are and may support coastal adaptation to sea-level rise that may be completed and expanded by a systematic review and additional research. We showed that current and future social science research can facilitate the implementation of adaptation and can contribute to shaping a socially desirable and feasible adaptation future in coastal areas. Beyond social sciences, other contributions could be considered, including artistic activities or literature fiction, which although not a social science can greatly help to raise awareness and disseminate science.

Based on this review, we suggest ways forward to amplify this effort and call for a strong development of coastal social science research. Specifically, we recommend to:

- expand the relatively limited scope of social research supporting coastal adaptation: this includes considering a wider variety of geographical contexts, coastal ecosystems and coastal management units;
- aim at better integrating emerging research areas identified above, while developing interdisciplinary studies linking social sciences with coastal science, climate services and the practice of coastal adaptation, which often remain focused on delivering climate and coastal hazard or risk geospatial datasets today.

Ultimately, these efforts could enable coastal communities, researchers and stakeholders to engage into broader transformations embracing issues such as social and economic development, mitigation of climate change as well as coastal ecosystem decline.

## Data Availability

The database on which this review relies is available in Supplementary Material S4.

## Supplementary material

For supplementary material accompanying this paper visit https://doi.org/10.1017/cft.2023.25. click here to view supplementary material
