# Supplementary figures and images for: Social science to accelerate coastal adaptation to sea-level rise
Source: Camb Prism Coast Futur. 2023 Aug 29;1:e37. doi: 10.1017/cft.2023.25 (PMC12337594; doi:10.1017/cft.2023.25)

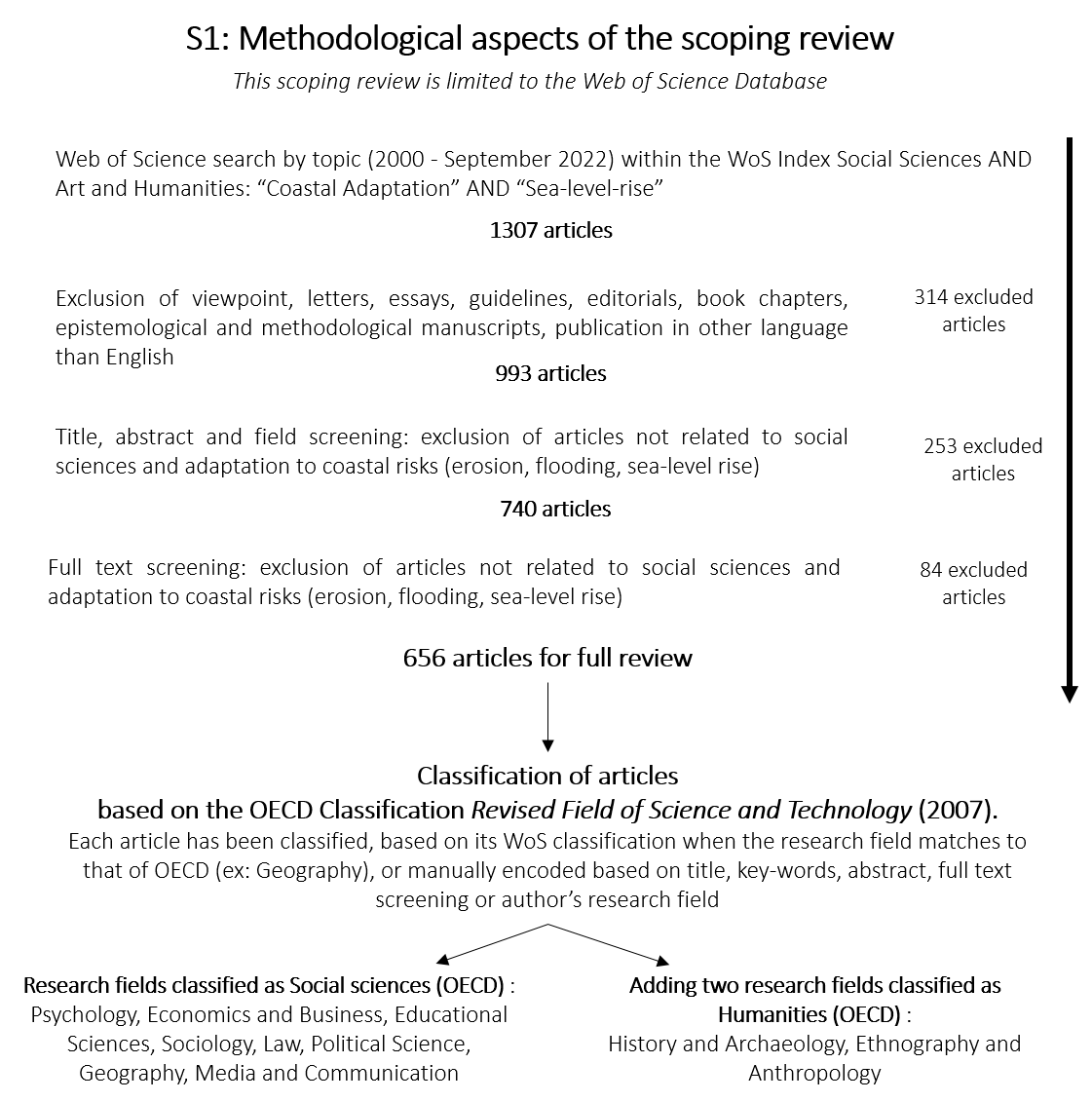

Supplement: Supplementary file 1 [file cftsup.zip › S2754720523000252sup001.png]

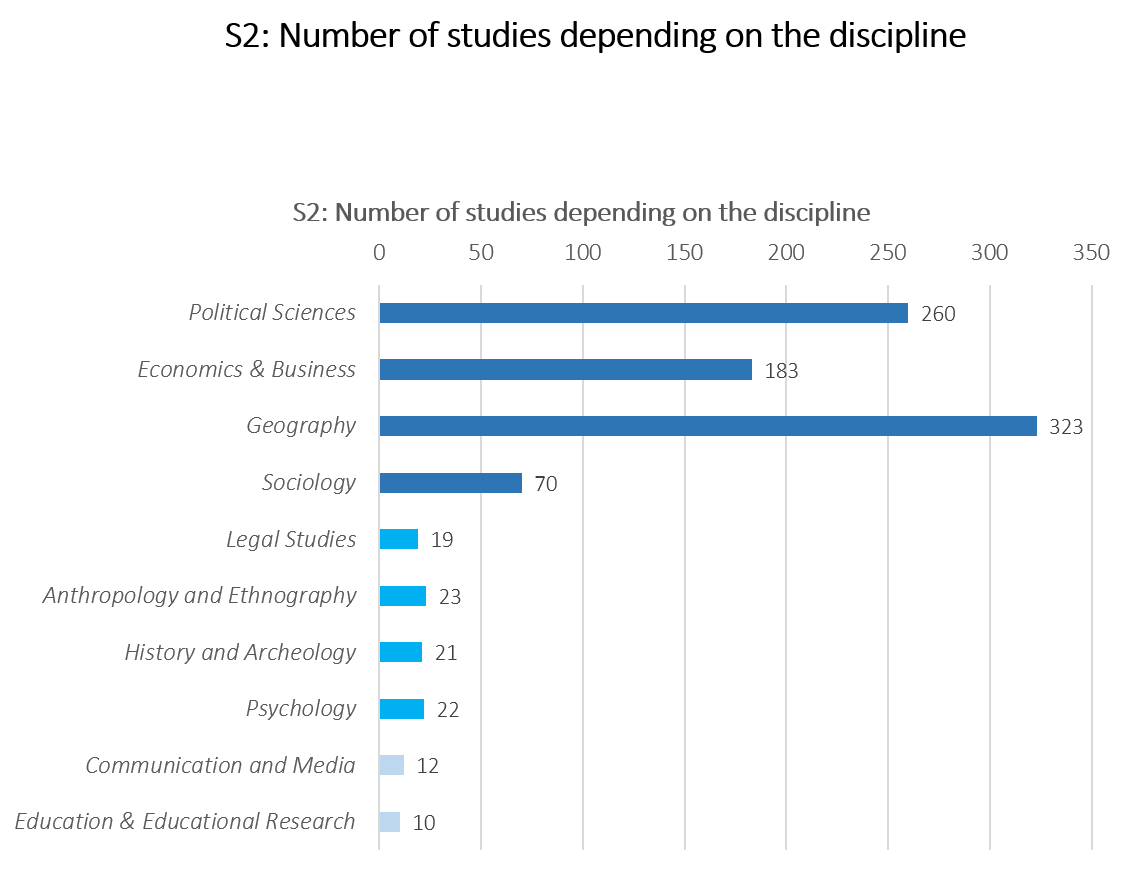

Supplement: Supplementary file 1 [file cftsup.zip › S2754720523000252sup002.png]

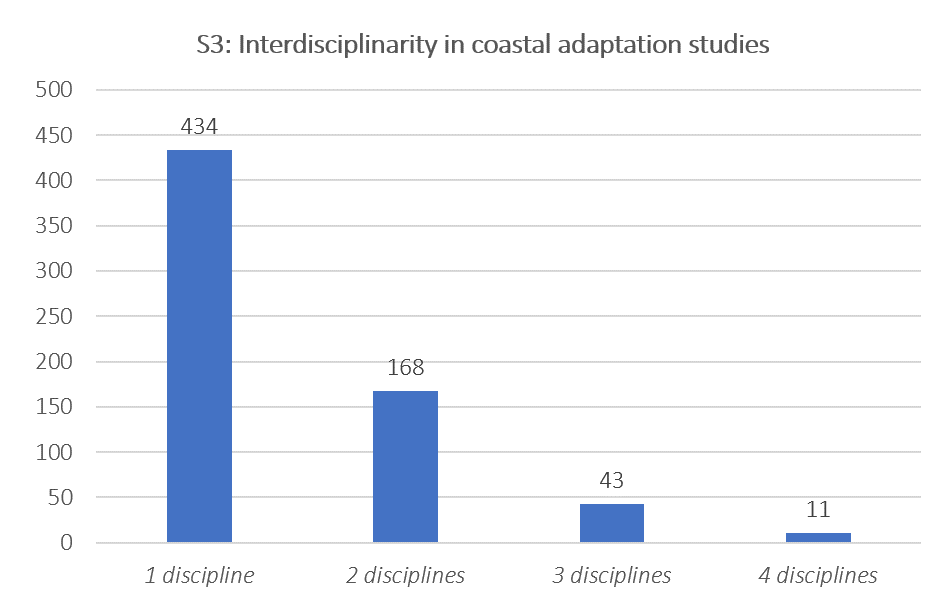

Supplement: Supplementary file 1 [file cftsup.zip › S2754720523000252sup003.png]
