# Supplementary material for: Social science to accelerate coastal adaptation to sea-level rise
Source: Camb Prism Coast Futur. 2023 Aug 29;1:e37. doi: 10.1017/cft.2023.25 (PMC12337594; doi:10.1017/cft.2023.25)
Supplement: Peer review history

# Reviewer report: Review: Social science to accelerate coastal adaptation to sea-level rise — R0/PR2

**DOI:** 10.1017/cft.2023.25.pr2

**Authors:** Annamaria Lammel

**Affiliations:** Universite Paris 8, France

Social science to accelerate coastal adaptation

Comments to Authors

This article addresses a very important topic: the adaptation of coastal regions to climate and environmental changes. It proposes to review the role of the different branches of social sciences that can enable sustainable coastal development. It presents several significant outcomes. Nevertheless, no scientific information is available regarding the review methodology.

Propositions

1/. Include social sciences as keywords.

2/. Summary: it is not clear what the article is about, and what is its methodology for getting the results presented in the abstract. What sort of review did the authors write? For instance, “We show that some bunches….” What does it mean we show, it is not precise!!!

3/. Impact statement: very general, requires a certain amount of precision.

4/. Page 2: Keywords are not the same as on the cover page, why?

5/. The term “accommodation” would need to be defined for Figure 1, Figure 2; page 3, line 75.

6/. Page 4, line 87 “We often assume that adaptation will be implemented in an optimal way.” Who are we? For optimism, maybe some references to psychology might be helpful. (e.g. Lammel, A., Dugas, E., & Guillen Gutierrez, E. (2012). L’apport de la psychologie cognitive à l’étude de l’adaptation aux changements climatiques: la notion de vulnérabilité cognitive. Vertigo, 12(1) ; Gardezi, M., & Arbuckle, J. G. (2020). Techno-optimism and farmers’ attitudes toward climate change adaptation. Environment and Behavior, 52(1), 82-105.)

7/. Page 4, line 79 (Figure 1; Bednar-Friedl et al., 2022) the reference in Figure 1 is different from the reference on page 3.

8/. Page 4, lines 89-102: This section is quite general and contains a few “errors”. It should be delayed or provide more accurate information, for example on the role of philosophy in the genesis of the social sciences. A distinction would have to be drawn between the humanities and the social sciences.

9/. Page 4, lines 104-105 “This review is based on a search, selection, and assessment of the existing literature using Web of Sciences (Table 1).” Methodological aspects of research, selection, and assessment have to be presented. Who encoded the papers? According to what criteria? Why solely WEB of Sciences? There are very different types of reviews, and there is a need to give specific information on the research protocol. (Various reviews: scoping review, systematic review, academic literature evidence map, systematic global inventory, etc.). For a method protocol of a systematic review, see for example Andreucci, M.B., de Vries, S., Marselle, M. R., Olszewska-Guizzo, A., Keune, H., O’Brien, L.,... and Lammel, A. (2019). Types and characteristics of urban green & blue spaces having an impact on human mental health and wellbeing: Methods Protocol, Knowledge assessment and synthesis. EKLIPSE.

10/. Page 7, lines 143 to 145, what are the criteria that apply to those classifications? How many scientific articles? The types of articles? The methodology?

11/. Figure 2 is unclear and does not present the relationship between the different adaptation options and the social sciences involved in these adaptive responses. This figure must be revised.

12/. Page 9, 205-206 “The number of studies relevant to these issues, while still limited, is growing (Merrill et al., 2018; Moser et al., 2019; Woodruff et al., 2020; Bisaro et al., 2020).” This phrase is somewhat ambiguous, what does it mean limited, but growing? I think this kind of quantitative commentary doesn’t make sense if the authors don’t give specific criteria.

13/. Page 10, lines 214-227 Legal science references are not really new, they do not appear to be an emerging research area.

14/. Page 12, line 294... I suggest adding: and provides knowledge as well!!

15/. Page 13, lines 307-309 As you gave only one example I propose to put this sentence in the conclusion part. “Here again, there is a need for more interdisciplinary research bringing together the different branches of social sciences supporting coastal adaptation.”

16/. Page 14, line 371 what is “long-term vision”, how many years, decades, centuries? How to integrate the different scenarios with a “long-term vision”?

17/. Figure 3 the Coastal adaptation circle and the coastal resilient development circle should be bidirectional.

18/. Page 16, 379-390 avoid some repetitions; 388-390 “Combined with artistic contributions, which although not a social science can greatly help to raise awareness and disseminate science, social sciences can now be considered as an essential part of science to enable coastal adaptation and resilient coastal development.” The role of art, even if I think is very important, it does not fit with the reviewed social sciences, maybe it should be integrated into “perspectives”.

19/. Pages 16, 17 maybe you should add a “perspective” section.

20/. Pages 35-38 Table 1: review of social science studies applied to coastal adaptation: If you have enough space, you could integrate more references (exhaustive) references in order to strengthen the review.

Table 1: Potential for enabling adaptation or resilient coastal development or potential for accelerate coastal adaptation (as in the title)?

# Reviewer report: Review: Social science to accelerate coastal adaptation to sea-level rise — R0/PR3

**DOI:** 10.1017/cft.2023.25.pr3

**Authors:** Sarah Vaughn

This artile seeks to outline the contributions of the social sciences to the study of coastal climate adaptation. The authors provide an overview of the social sciences as it relates to a broad field of discplines and find that the discpline of economics and political science have made most of the contributions to date. The paper, however, conflates literature that is in the applied social sciences with the interperative and theory-driven social sciences scholarship. Consequently, the authors fail to review a wide array of literature, particularly in anthropology and area studies, that has made contributions to the topic of coastal climate adaptation. Also, the authors tend to attribute such broad themes as “colonialism” and “inequality” to being resolutely “social science” topics, while also citing literature on these themes that have very little to do with coastal climate adaptation. I’d encourage the authors to more clearly engage distinctions between applied and interperative/theory-driven scholarship in the social sciences on coastal climate adaptation. In addition, I would suggest that the authors add a “coda” section that offers an analysis as to why/how the existing governance frameworks of the IPCC perpetuate an overemphasis on applied scholarship; and on the other hand, blindly encourage the narrowing of social science literature around themes, such as ‘justice’ and ‘equity,’ in the the study of coastal climate adpatation.

# Editor report: Recommendation: Social science to accelerate coastal adaptation to sea-level rise — R0/PR4

**DOI:** 10.1017/cft.2023.25.pr4

**Affiliations:** Università degli Studi di Ferrara, Italy

No accompanying comment.

# Editor report: Decision: Social science to accelerate coastal adaptation to sea-level rise — R0/PR5

**DOI:** 10.1017/cft.2023.25.pr5

**Affiliations:** Geography, Cambridge University, United Kingdom of Great Britain and Northern Ireland

No accompanying comment.

# Reviewer report: Review: Social science to accelerate coastal adaptation to sea-level rise — R1/PR7

**DOI:** 10.1017/cft.2023.25.pr7

**Authors:** Annamaria Lammel

**Affiliations:** Universite Paris 8, France

Dear Author,

The article is now ready to be published. The article’s quality was improved by the modifications. The importance of social sciences in coastal adaptation to sea-level rise is fully demonstrated by it.

# Editor report: Recommendation: Social science to accelerate coastal adaptation to sea-level rise — R1/PR8

**DOI:** 10.1017/cft.2023.25.pr8

**Affiliations:** Università degli Studi di Ferrara, Italy

No accompanying comment.

# Editor report: Decision: Social science to accelerate coastal adaptation to sea-level rise — R1/PR9

**DOI:** 10.1017/cft.2023.25.pr9

**Affiliations:** Geography, Cambridge University, United Kingdom of Great Britain and Northern Ireland

No accompanying comment.
